# Intronic mutation of the *VHL* gene associated with central nervous system hemangioblastomas in two Chinese families with Von Hippel–Lindau disease: case report

**DOI:** 10.1186/s12881-020-01126-7

**Published:** 2020-10-01

**Authors:** Zhen Liu, Jingcheng Zhou, Liang Li, Zhiqiang Yi, Runchun Lu, Chunwei Li, Kan Gong

**Affiliations:** 1 Department of Neurosurgery, Peking University First Hospital, No. 8 Xishiku Street, Xicheng District, Beijing, 100034 China; 2 Department of Urology, Peking University First Hospital, Beijing, 100034 China

**Keywords:** Central nervous system, Hemangioblastoma, Von Hippel–Lindau, Intronic mutation, Case report

## Abstract

**Background:**

Central nervous system (CNS) hemangioblastomas are the most frequent cause of mortality in patients with Von Hippel–Lindau (VHL) disease, an autosomal dominant genetic disease resulting from germline mutations in the *VHL* tumor suppressor gene, with most mutations occurring in the exons. To date, there have been no reports of CNS hemangioblastoma cases related to pathogenic variants in intron 2 of *VHL*, which encodes a tumor suppressor protein (i.e., pVHL) that regulates hypoxia-inducible factor proteins.

**Case presentation:**

We report the presence of a base substitution of c.464-1G > C and c.464-2A > G in the intron 2 of *VHL* causing CNS hemangioblastomas in six patients with VHL from two Chinese families. The clinical information about the two pathogentic variants has been submitted to ClinVar database. The ClinVar accession for NM_000551.3(VHL):c.464-1G > C was SCV001371687. This finding may provide a new approach for diagnosing and researching VHL-associated hemangioblastomas.

**Conclusions:**

This is the first report of a pathogenic variant at intron 2 in VHL-associated hemangioblastomas. Gene sequencing showed that not only exonic but also intronic mutations can lead to the development of CNS hemangioblastomas.

## Background

Von Hippel–Lindau (VHL) disease is an autosomal dominant genetic disease resulting from germline mutations in the *VHL* tumor suppressor gene on chromosome 3 (3p25–26) within a 10 kb region. This gene consists of two introns and three exons and encodes a tumor suppressor protein (i.e., pVHL) that regulates hypoxia-inducible factor (HIF) proteins [1–3]. The main manifestation of VHL disease in the central nervous system (CNS) is hemangioblastoma (HGB). Some studies have shown that partial deletions are associated with a high risk of developing a large number of CNS HGBs. However, most mutations occur in the exons [4]. To date, no cases of intron 2 of VHL associated with CNS HGBs have been reported. Here, we report the cases of six patients with VHL-associated HGBs from two Chinese families with two types of intronic pathogenic variant in *VHL*.

## Case presentation

The study was approved by the Ethics Committee of Peking University First Hospital and conformed to the Helsinki Declaration.

**Fig. 1:**
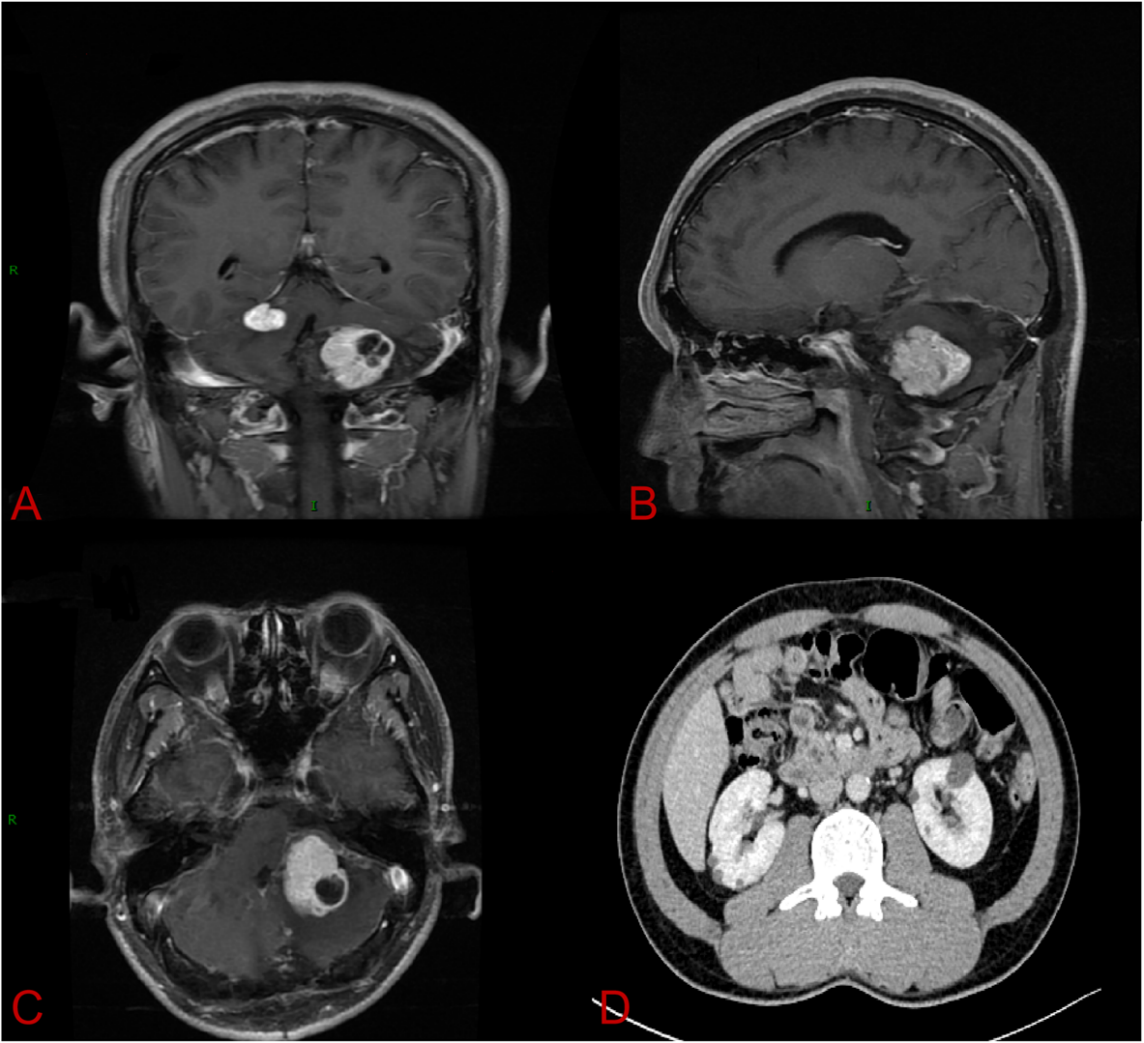
**a–c**Contrast-enhanced magnetic resonance imaging (MRI) of the cerebellum hemangioblastomas in the 40-year-old male patient with VHL. **d.** Abdominal computed tomography (CT) scan with contrast demonstrated multiple cysts in the pancreas and kidneys in the 40-year-old male VHL patient

A 40-year-old male was admitted to Peking University First Hospital in 2018 with headache and dizziness that had been ongoing for 2 months. Notably, he had undergone surgical resection for cerebellar HGBs in 2015. When he was admitted in 2018, contrast-enhanced magnetic resonance imaging (MRI) of the cerebellum (3.0 T; Signa Excite HD 3.0 T; GE Healthcare, Chicago, IL, USA) showed multiple lesions in the cerebellum with obvious enhancement, highly suggestive of HGBs (Figs. 1a–c). In addition, abdominal computed tomography (CT) scanning with contrast revealed multiple cysts in the pancreas and kidneys (Fig. 1d). Because of the obvious occupy effect and symptoms, he underwent operation again. Postoperative pathology demonstrated that both lesions resected from the cerebellum were HGBs. The patient’s symptoms improved after surgery, and at two-year follow-up (FU), his condition was found to be stable with no recurrence. Five of his family members from three generations has also undergone surgical resections for CNS-related HGBs. The patient provided written informed consent to participate in this study and for publication of this case report.

**Fig. 2:**
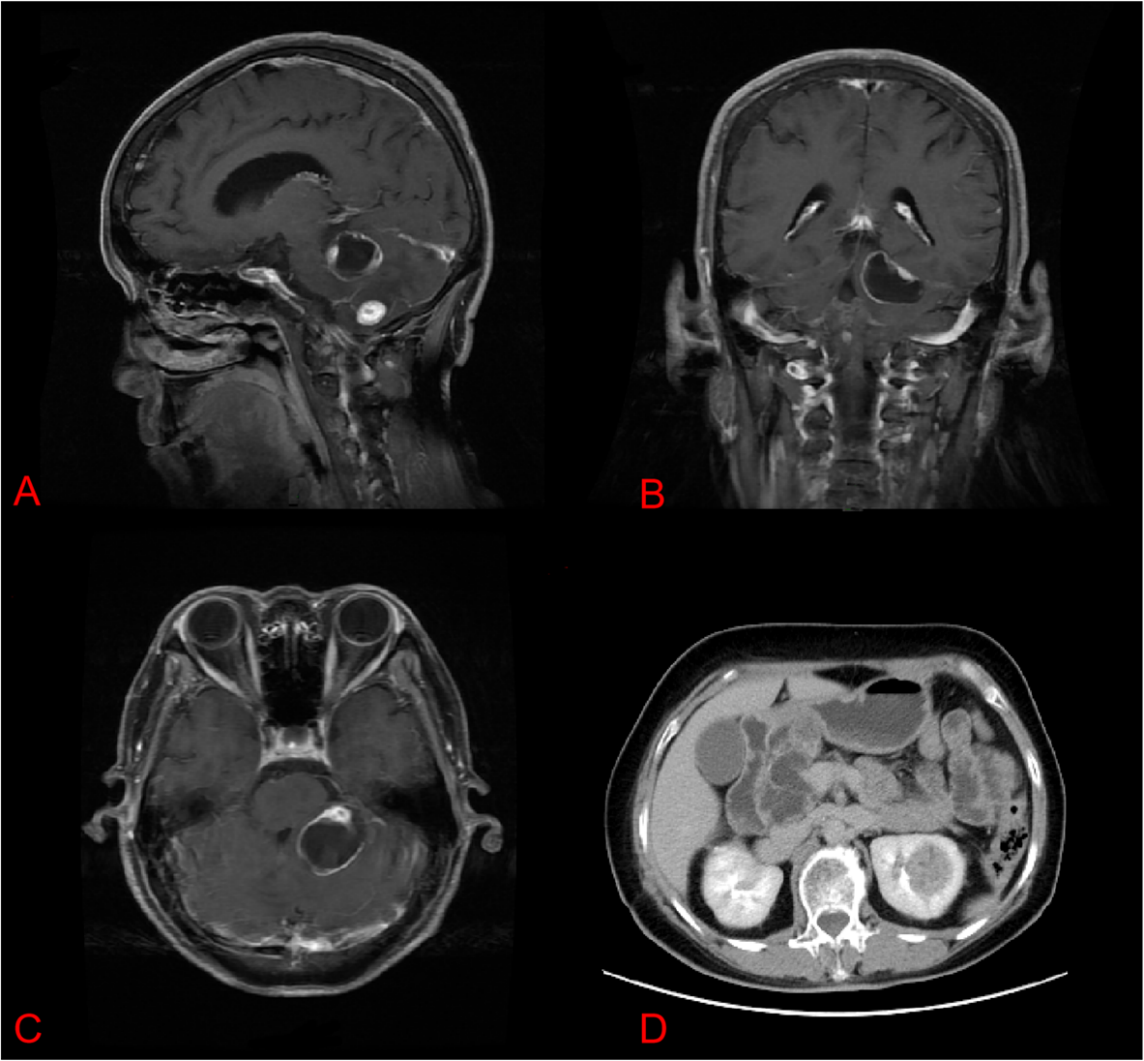
**a–c** Contrast-enhanced magnetic resonance imaging (MRI) of the cerebellum hemangioblastomas in the 58-year-old female patient. **d.** Abdominal computed tomography (CT) scan with contrast demonstrated renal cell cancer and multiple renal and pancreatic cysts in the same patient

A 58-year-old female was admitted to our hospital with symptoms similar to those of the male patient. Contrast-enhanced MRI showed multiple HGBs in the cerebellum with an obvious occupy effect (Fig. 2a–c). In addition, contrast-enhanced abdominal CT showed renal cell cancer and multiple renal and pancreatic cysts (Fig. 2d). She underwent surgical resection for HGBs in the bilateral cerebellum, and her symptoms improved. At two-year FU, her condition was stable with no recurrence.

Both patients and their family members who met the clinical diagnostic criteria underwent genetic testing. Clinical, genetic, and relevant imaging data were analyzed.

Genomic DNA was extracted from peripheral blood using a QIAamp DNA Blood Mini Kit (QIAGEN, Hilden, Germany). Three coding exons and their flanking intronic regions were amplified by polymerase chain reaction (PCR) using previously described primers [5]. Direct sequencing was performed to screen missense and splicing mutations. Large exon deletions were screened using a multiplex ligation-dependent probe amplification kit (P016-C2; MRC Holland, Amsterdam, the Netherlands) and confirmed by real-time quantitative PCR with primers described by Ebenazer et al. [6]. The reaction conditions and primers used for amplification were as previously described [7].

**Fig. 3:**
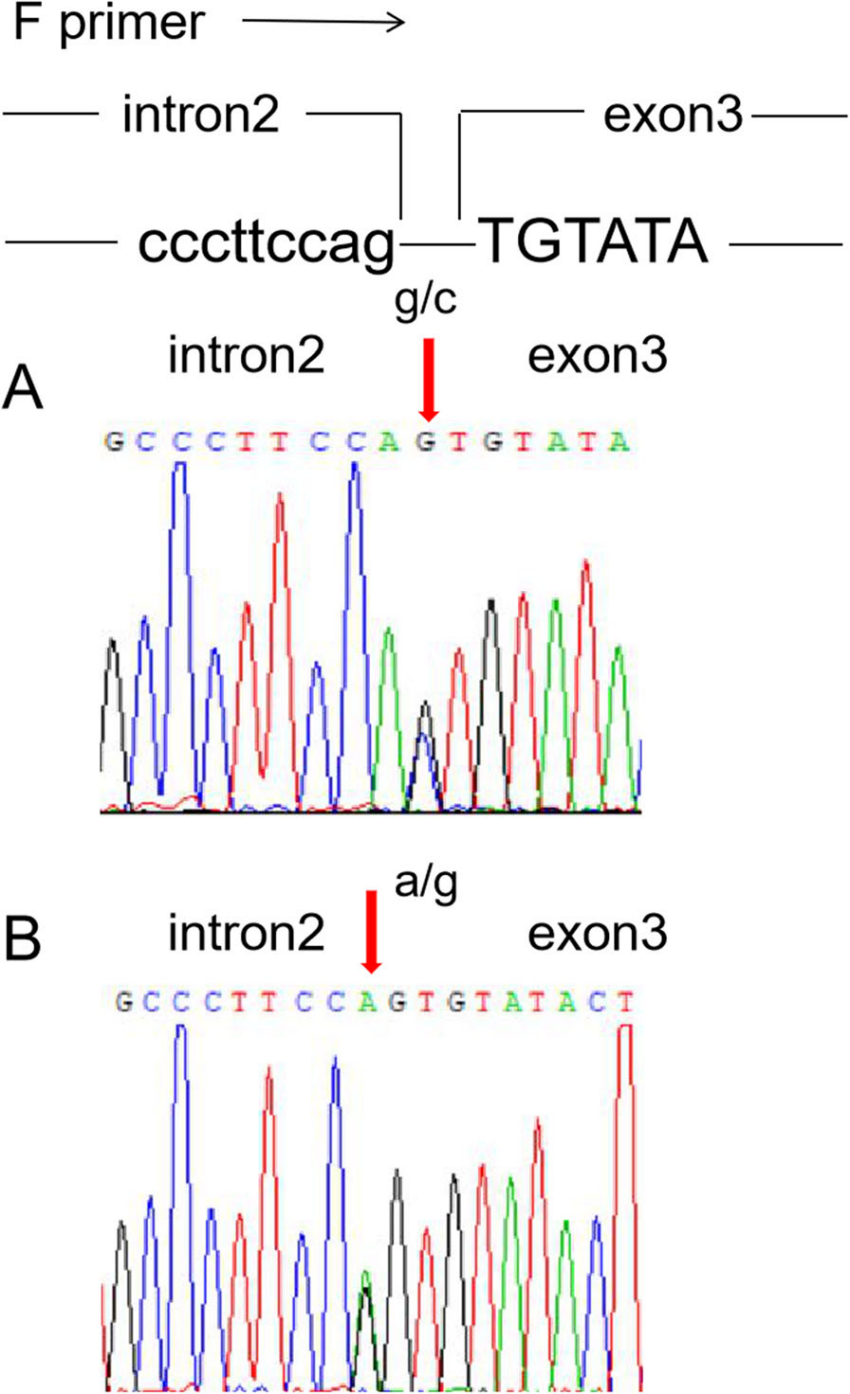
Fragment of chromatogram showing the sequence of intron 2 and exon 3 of VHL gene in proband. Sequences amplified by forward primer (upper picture). **a.** Heterozygous c.464-1G > C point mutation in *VHL* (red arrow). **b.** c.464-2A > G point mutation (red arrow)

**Fig. 4:**
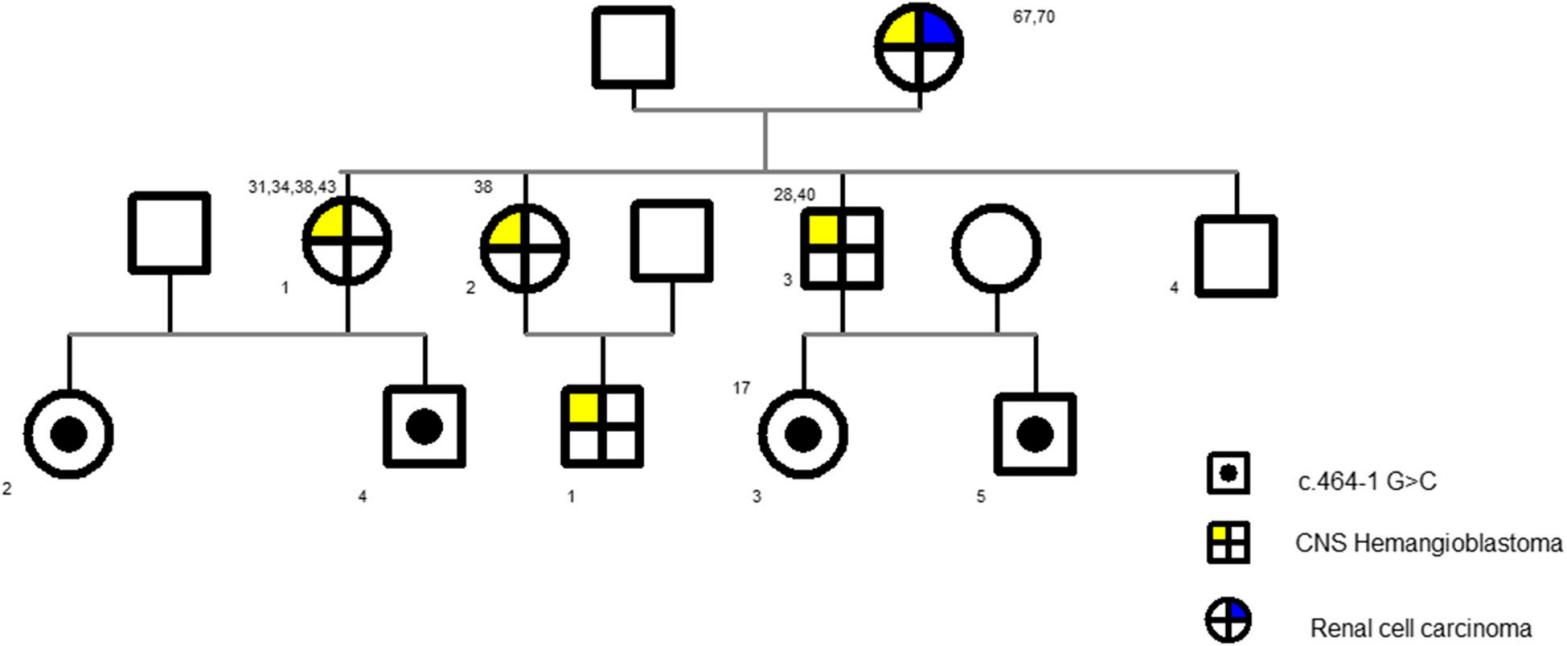
Family tree of the patients with c.464-1G > C point mutation. Three-generation pedigree of the patient family showing that the synonymous VHL variant c.464-1G > C segregates with affected family members. The number on the upper left corner of each patient with VHL-associated HGBs indicates the age of each operation for CNS HGBs

Gene sequencing revealed a heterozygous germline mutation (NM_000551.3(VHL):c.464-1G > C) in the splice site in intron 2 of *VHL* in the DNA from the peripheral blood of the male patient and nine of his family members (male 4, female 5) (Fig. 3a). In total, five of these nine patients from three generations underwent a total of nine surgical resections for CNS-related HGBs, with an average of 1.8 ± 1.3 surgeries (range: 1–4). All resected lesions were located in the cerebellum. Figure 4 shows the genetic investigation used in this study. In the first, second, and third generations, the ages at which the first operation for CNS HGBs was performed were 67, 32.3, and 17 years, respectively. Notably, the age at which the first operation for CNS HGBs was performed was younger in the children (younger generation) than in their parents (older generation). Additionally, only the mother of the male patient with the NM_000551.3(VHL):c.464-1G > C variant was diagnosed with renal cell carcinoma and underwent left nephrectomy. Another intronic mutation in *VHL* occurred in NM_000551.3(VHL):c.464-2A > G in the 58-year-old female patient (Fig. 3b), but none of her family members showed any abnormal changes in *VHL*. The clinical information about the two pathogentic variants has been submitted to ClinVar database. The ClinVar accession for NM_000551.3(VHL):c.464-1G > C was SCV001371687.

## Discussion and conclusions

Some studies reported a germline mutation in intron 1 of *VHL*. This intronic mutation at the splice site occurs in various VHL-related diseases, such as erythrocytosis (NM_000551.4(VHL):c.340 + 770 T > C), renal cancer, or even tongue cancer (VHL c.340 + 5G > C) [8, 9]. However, the pathogenic mechanism and importance of this mutation remain unclear. The most likely cause of this condition is abnormal splicing. Lenglet et al. [8] also assumed that this intronic mutation (NM_000551.4(VHL):c.340 + 770 T > C) affects exonic splicing, resulting in the failure to produce a VHL protein. Genetic mutations also cause pVHL inactivation, resulting in dysfunction in the regulation of proteolytic degradation of HIF proteins [10]. In addition, uncontrolled HIF expression increases the expression of a wide range of target genes involved in angiogenesis, proliferation, and metabolism, including the vascular endothelial growth factor and C-X-C motif chemokine receptor 4. It is well-known that overexpression of VEGF is a leading factor in tumor angiogenesis, particularly for HGBs [11, 12]. In addition, several researchers have suggested that nearly one-third of HGBs are related to VHL disease [13]. Ong and Whaley reported the NM_000551.3(VHL):c.464-1G > C and NM_000551.3(VHL):c.464-2A > C mutations, respectively [14, 15]. However, both studies only described the intronic mutation in sporadic renal cell carcinoma. Neither study could determine whether the intronic variant was pathogenic. A query of the ClinVar database revealed that our research is the first report of a germline mutation at c.464-1G > C and c.464-2A > C in VHL-associated HGBs. This is also the first report of a pathogenic intron variant (NM_000551.3(VHL):c.464-1G > C) in a pedigree with VHL disease. Sequencing results showed that a base substitution of c.464-1G > C and c.464-2A > G in the intron 2 splice donor site of *VHL* could result in abnormal splicing. The family history of these patients with HGB was accordance with strong evidence showing that cosegregation with disease in multiple affected family members in a gene definitively known to cause the disease. According to the American College of Medical Genetics and Genomics standards and guidelines, we found that both variants were pathogenic [16]. According to previous research, there is increasing evidence that CNS HGBs are the most frequent cause of mortality in patients with VHL disease [17, 18]. However, some studies reported that approximately 20% of patients have VHL disease resulting from a de novo mutation and have no family history [19]. Thus, not all CNS HGB cases are screened for germline *VHL* mutations and the rate may be underestimated. Notably, both patients mentioned above, the female patient with c.464-2A > G and the male patient’s mother with c.464-1G > C, exhibited a nervous system manifestation as the initial symptom caused by CNS HGBs. Both patients underwent surgical treatment for renal cell carcinoma 2–3 years after CNS surgery. Hence, detecting molecular changes has several implications for the clinical management of these patients who are suspected to have VHL disease with CNS HGBs as the initial pathogenic manifestations. To our knowledge, this is the first report of a germline mutation in intron 2 in VHL-associated HGBs. The gene sequencing results showed that not only the exon region but also the intronic mutation could lead to the development of CNS HGBs. When no mutation can be detected in the exons, the possibility of intronic mutation cannot be ignored. In addition, pedigree analysis demonstrated that the age at which the first operation was performed was younger in the children than in their parents. Some researchers have suggested that the regularity with which this occurs is associated with genetic anticipation. The reasons for this phenomenon are considered to be related to telomere shortening in the offspring [20].

In this study, it was found that not only exonic mutations but also intronic mutations (c.464-2A > G and c.464-1G > C) in *VHL* play a pivotal role in the occurrence of CNS HGBs, which may provide a new approach for the diagnosis and research of VHL-associated HGBs.

## Data Availability

The raw datasets generated and analyzed during the current study are not publicly available because it is possible that individual privacy could be compromised but are available upon reasonable request with fulfillment of Materials Transfer Agreement. To request the datasets, please contact the corresponding authors (Liang Li doctzyf@126.com). The dataset corresponding to the NM_000551.3(VHL):c.464-1G > C and NM_000551.3(VHL):c.464-2A > G gene can be found in NCBI under the accession number LOC107303340 (https://www.ncbi.nlm.nih.gov/gene/?term=LOC107303340). The c.464-1G > C variation was submitted to ClinVar database (https://www.ncbi.nlm.nih.gov/clinvar/) and the ClinVar accession number SCV001371687 assigned to it.

## Abbreviations

CNS: Central nervous system
HGBs: Hemangioblastomas
VHL: Von Hippel–Lindau
HIF: Hypoxia-inducible factor
VEGF: Vascular endothelial growth factor
